# Mitochondrial Genomes Yield Insights into the Basal Lineages of Ichneumonid Wasps (Hymenoptera: Ichneumonidae)

**DOI:** 10.3390/genes13020218

**Published:** 2022-01-25

**Authors:** Boying Zheng, Yuanyuan Han, Ruizhong Yuan, Jingxian Liu, Pu Tang, Cornelis van Achterberg, Xuexin Chen

**Affiliations:** 1 State Key Laboratory of Rice Biology, Zhejiang University, Hangzhou 310058, China; zhengboyingg@163.com (B.Z.); yyhan6@zju.edu.cn (Y.H.); monareason@zju.edu.cn (R.Y.); kees@vanachterberg.org (C.v.A.); xxchen@zju.edu.cn (X.C.); 2 Institute of Insect Sciences, College of Agriculture and Biotechnology, Zhejiang University, Hangzhou 310058, China; 3 Department of Entomology, College of Plant Protection, South China Agricultural University, Guangzhou 510642, China; jingxianliu@foxmail.com; 4 Zhejiang Provincial Key Laboratory of Biology of Crop Pathogens and Insects, Zhejiang University, Hangzhou 310058, China; 5 Ministry of Agriculture Key Lab of Molecular Biology of Crop Pathogens and Insects, Zhejiang University, Hangzhou 310058, China

**Keywords:** mitochondrial genome, gene rearrangement, ichneumonid wasps, Eucerotinae, Xoridinae, phylogeny

## Abstract

We obtained four mitochondrial genomes of *Odontocolon* sp., *Xorides funiuensis*, *Euceros kiushuensis* and *Euceros serricornis*, which represent the first two representatives from subfamily Eucerotinae and Xoridinae (Ichneumonidae), respectively. All of the 4 newly sequenced mitochondrial genomes contain 13 protein-coding genes (PCGs) and most 24 RNA genes. Furthermore, they all have novel tRNA rearrangement patterns comparing with published mitogenomes in Ichneumonidae. For the tRNA cluster *trnI-trnQ-trnM*, *X. funiuensis* is shuffled as *trnM-trnI-trnQ* with *trnQ* inversed, while *Odontocolon* sp. with a remote translocation of *trnK*, shuffling as *trnI-trnM-trnQ*. *E. kiushuensis* and *E. serricornis* shared the same cluster *trnQ-trnY-trnW-trnC*. Finally, we reconstructed the phylogenetic relationships among the sequenced subfamilies of Ichneumonidae based on nucleotides and amino acids sequences of 13 PCGs in mitochondrial genomes, and the results of both the maximum likelihood and Bayesian inference analyses highly support that Eucerotinae is the basal ichneumonid lineage rather than Xoridinae.

## 1. Introduction

The family Ichneumonidae (Insecta: Hymenoptera) is one of the most species-rich animal groups on the Earth, with around 25,000 species described according to Taxapad (2016) [1,2,3,4]. They are also the largest group of parasitic wasps, with a mega diversity of specialized characteristics and habits. Their hosts include many holometabolous insects, especially Coleoptera and Lepidoptera, and some lineages even associated with spiders [3]. Different wasp species can oviposit on the larval, pupal or adult stages of hosts and subsequently kill the hosts as food supply for themselves [5]. Therefore, Ichneumonidae are economically important as they play a great role in maintaining the balance in nature and the biological control in agricultural pests [3]

Ichneumonidae are grouped into 42 subfamilies at present, and the phylogenetic relationships among subfamilies remain controversial [3,5]. Most subfamilies of Ichneumonidae are included in three high-level groupings Pimpliformes, Ichneumoniformes and Ophioniformes, suggested by both morphological and molecular characteristics [6,7,8]. However, the evolutionary placement of several highly specialized subfamilies on morphology and habits, such as Eucerotine and Xoridinae, are more likely to be contentious [7,8,9,10,11,12]. Quicke et al. (1999) pointed out that Xoridinae is considered as a basal lineage of Ichneumonidae according to a 28S region rDNA sequences analysis, which was supported by some other studies [9,12]. However, Bennett et al. (2019) employed several different analyses for 42 currently recognized subfamilies in Ichneumonidae and refuted that Xoridinae is the sister to all other Ichneumonidae [8]. The position of Eucerotinae is uncertain, once considered to be within Tryphoninae (paraphyletic in that analysis), near to Ophioniformes or closely related with Ichneumoformes [7,8,9,10,11].

Animal mitochondrial DNA is maternally inherited, non-recombining with an elevated mutation rate compared to nuclear DNA and it was thought to be the most popular marker in phylogenetic analysis of insects for a period [13,14,15,16,17,18]. The typical insect mitochondrial genome is circular, being ~16 kb in length and normally containing 13 protein-coding genes (PCGs), 22 transfer RNA (tRNA) genes, 2 ribosomal RNA (rRNA) genes and a A + T-rich region (control region, CR) [13,14]. Mitochondrial genomes in the Hymenoptera exhibit many unique features, such as frequent gene rearrangement [19], high substitution rates [20] and a strong base composition bias [21]. However, the sequencing of mitochondrial genomes of Ichneumonidae is very limited. So far, only 11 complete or partial mitogenomes of ichneumonid wasps were reported in the GenBank (up to July 30, 2021), which include only 5 subfamilies in all of the 42 extant subfamilies within Ichneumonidae [3].

Therefore, the mitogenomes from the remaining subfamilies of Ichneumonidae are warranted to be sequenced for the understanding of the mitochondrial characters and its phylogenetic relationships within Ichneumonidae. In this study, to increase the number of ichneumonid’s mitogenomes and detect the placement of Eucerotine and Xoridinae within Icheneumonidae, we sequenced and assembled four mitochondrial genomes which act for the first two representatives of subfamilies Eucerotinae and Xoridinae of Ichneumonidae, respectively. We described and compared the main features for these mitogenomes and investigated the phylogenetic relationships within Ichneumonidae based on 14 mitochondrial genomes, including the 4 newly sequenced mitogenomes as 2 mitogenomes from the Braconidae of the outgroup.

## 2. Materials and Methods

### 2.1. Species Identification and DNA Extraction

The voucher specimens for 4 newly sequenced mitochondrial genomes were identified by Dr. Jingxian Liu based on their morphology (Table S1). All specimens were preserved in 100% ethanol and stored at 4 °C before DNA extraction. Each individual sample was used for whole genomic DNA extraction separately without destroying samples using DNeasy® Blood and Tissue Kit (Qiagen, Hilden, Germany) [22,23]. Voucher specimens are deposited in Institute of Insect Sciences, Zhejiang University (Voucher specimen numbers: ZJUHW_202000017-ZJUHW_202000020, Table S1).

### 2.2. Next-Generation Sequencing and Assembly

All libraries were constructed using VAHTS^TM^ Universal DNA Library Prep Kit. Indexed libraries were sequenced employing Illumina NovaSeq sequencer (150 bp pared-end) of Novogene.

The DNA extraction and mitogenome assembly were conducted following a modified version of the pipeline. FastQC was used to check the quality of the data, and Trimmomatic was used to trim adaptors and indices with default parameters [15]. The target mitochondrial reads were filtered out using BLAST (BLASTn with E value: 1 × 10^−5^) against a reference dataset containing published Ichneumonidae mitochondrial genomes [24]. Reads of mitochondrial genomes were assembled by Spades v3.0 with default parameters [25].

### 2.3. Mitochondrial Genes Annotation

Annotation of assembled contigs was performed using the mitogenome annotation web server (http://mitos.bioinf.uni-leipzig.de/index.py, accessed date: January 2020). Start and stop codons of protein-coding genes were manually adjusted in Geneious Prime v11 by referencing to the published mitogenomes of Ichneumonidae. The online tRNAscan-SE service (http://lowelab.ucsc.edu/tRNAscan-SE/, accessed date: January 2020) was used to confirm locations of tRNA genes [26]. The obtained mitochondrial genomes were submitted to GenBank (GenBank Accession numbers: MT252850-MT252853).

### 2.4. Comparative Analysis of the Mitochondrial Genomes from Ichneumonidae

Characteristics of mitochondrial genomes of sequenced species in Ichneumonidae were analyzed, including gene arrangements, base compositions, codon usages and evolutionary rates (Ka, Ks and Ka/Ks) of PCGs. Gene rearrangements were analyzed by comparing with the ancestral mitochondrial genome. The base composition was obtained by MEGA v7.0 [27]. The AT-skew and GC-skew were calculated according to the following formulae: AT-skew = (A% − T%)/ (A% + T%) and GC-skew = (G% − C%)/ (G% + C%) [28]. The relative synonymous codon usage (RSCU) of all protein-coding genes was calculated in Geneious Prime v11. Synonymous (Ks) and non-synonymous (Ka) substitution rates of PCGs were calculated using DnaSP v6.12.03 [29].

### 2.5. Phylogenetic Analysis

Mitochondrial genomes of 12 species representing the family Ichneumonidae, including 4 newly-sequencing species, were used to construct phylogenetic relationship of Ichneumonidae (Table 1). *Zele chlorophthalmus* and *Cotesia vestalis*, both from Braconidae, were chosen as outgroups (Table 1).

The PCGs were aligned using G-INS-i algorithms implemented in MAFFT v7.464 [30,31]. Mrbayes v3.2.7a [32] and IQtree v2 [33] were independently used to reconstruct the phylogenetic tree based on amino acids (AA) and nucleotides (NU) sequences of 13 protein-coding genes, respectively. For Bayesian inference analysis (BI), the best partition schemes of substitution models for the matrix were searched in PartitionFinder v2 with model selection = BIC and Branch lengths = unlinked between different subsets (Table S2) [34]. The best model schemes were used for 100 million generations, with tree sampling occurring every 1000 generations and a burn-in of 25% of the trees in Mrbayes to phylogenetic analysis. In maximum likelihood (ML) analysis, ModelFinder (software within IQtree) was used to search best models (Table S3) for different individual partitions to construct ML trees with 1,000 bootstrap replicates.

## 3. Results and Discussion

### 3.1. General Features of Mitochondrial Genomes

Four partial mitochondrial genomes from Ichneumonidae have been sequenced, including *Odontocolon* sp. (MT252850) and *X. funiuensis* (MT252851) from Xoridinae, *E. kiushuensis* (MT252852) and *E. serricornis* (MT252853) from Eucerotinae (Table 1). The mitochondrial sequences of *Odontocolon* sp., *X. funiuensis*, *E. kiushuensis* and *E. serricornis* are 13,321 bp, 14,939 bp, 16,331 bp and 15,787 bp in length, respectively (Table 2). The control region (CR) of all the species failed to be assembled owing to its natural characteristics of high duplications of base A and T. *X. funiuensis* and *E. kiushuensis* contain 37 typical genes. The *trnI* failed to be assembled in *E. serricornis*. *Odontocolon* sp. had 33 genes, with the *trnK*, *trnV* and *rrnS* genes being absent.

### 3.2. Base Composition, Codon Usage and Evolutionary Rate

The four mitogenomes are partial so that we solely calculated A + T content of 13 PCGs in base composition evaluation. The (A + T) % of all PCGs is 78.60% for *Odontocolon* sp., 82.35% for *X. funiuensis*, 83.99% for *E. kiushuensis* and 83.53% for *E. serricornis* (Table 2). This base composition is usual in Ichneumonidae, ranging from 84.01% (both *Enicospilus* sp. and *Venturia canescens*) to 78.26% (*Hypsicera* sp.) (Table 2). All of the AT-skews are negative, similar to other ichneumonid species (Table 2). The GC-skews of *Odontocolon* sp. (−0.1009) and *X. funiuensis* (-0.1180) are also negative as reported for most Hymenoptera, even insects (Table 2) [1,35], while those of *Euceros* spp. (0.0782 and 0.0537) and *Enicospilus* sp. (0.0330) are positive as reported for the most species of the sister-family Braconidae (Ichneumonoidea), which is an unusual feature of ichneumonid mitogenomes [35].

**Figure 1:**
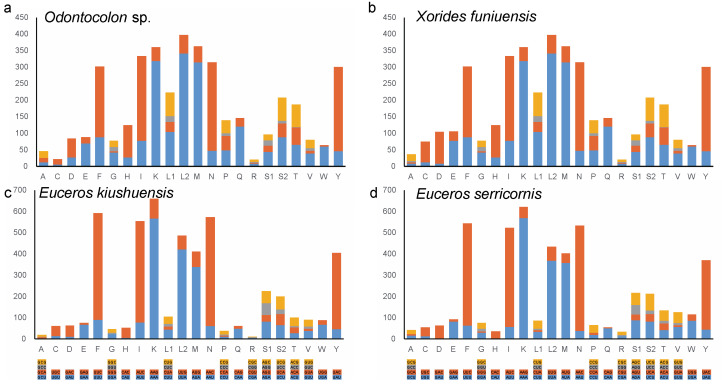
Relative synonymous codon usage (RSCU) in the mitochondrial protein-coding genes (PCGs) that four mitogenomes of Ichneumonidae sequenced in this study. The y-axis represents the counts for condon and animo acid usage. The terminal codon is not given. (**a**) The RSCU counts of *Odontocolon* sp.; (**b**) The RSCU counts of *X. funiuensis*; (**c**) The RSCU counts of *E. kiushuensis*; (**d**) The RSCU counts of *E. serricornis*.

The average relative synonymous codon usage (RSCU) counts of these four species were calculated (Figure 1). All possible combinations of synonymous codons representing amino acids are present as invertebrate mitochondrial genes. Both species in the subfamily Xoridinae and Eucerotinae share a similar RSCU style. Amino acids L (Leu), especially for L2, possess the highest RSCU value in Xoridinae. However, K (Lys) own the highest RSCU value, and the RSCU value of L (Leu) (containing L1 and L2) is distinctly decreased in Eucerotinae. F (Phe) are the more frequently encoded amino acids in Eucerotinae than Xoridinae (Figure 1).

**Figure 2:**
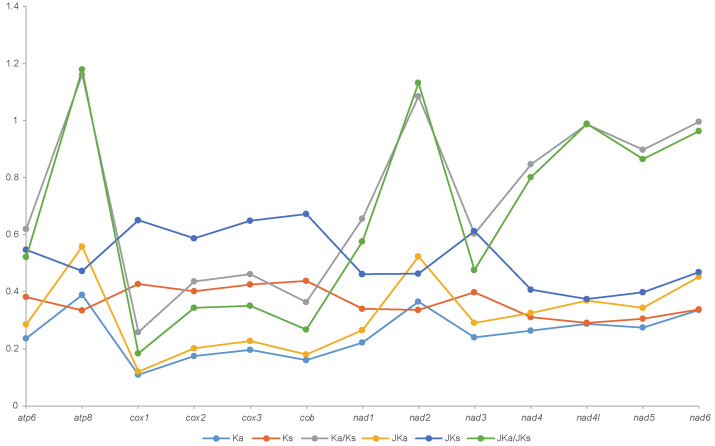
Synonymous (Ka) and non-synonymous substitutional (Ks) rates and the ratios of Ka/Ks of 13 protein coding genes (PCGs) in mitochondrial genomes of all sequenced species in Ichneumonidae. JKa, JKs and JKa/JKs are the values corrected by the Jukes–Cantor correction (Jukes and Cantor, 1969).

The evolutionary rates (Ka, Ks and Ka/Ks) of PCGs vary considerably among genes (Figure 2; Table S4). In Ichneumonidae, the Ka values of 13 PCGs range from 0.11008 (*cox1*) to 0.38877 (*atp8*), and Ks values range from 0.29076 (*nad4l*) to 0.43764 (*cob*). After being corrected by the Jukes–Cantor correction [37], the Ka and Ks values were magnified sharply, with the minimum and maximum of JKa up to 0.11948 (*cox1*) and 0.67294 (*atp8*) and of JKs up to 0.37384 (*cox1*) and 0.55786 (*atp8*), respectively (Figure 2; Table S4). In all tested PCGs, *atp8* and *nad2* have the highest Ka/Ks (1.16061, 1.08557) and JKa/JKs (1.17988, 1.13260), supporting that *atp8* and *nad2* are the most divergent PCGs in Ichneumonidae (Figure 2; Table S4). Genes *cox1* and *cob* have the lowest evolutionary rates (*cox1*: Ka/Ks = 0.25750, JKa/JKs = 0.18374; *cob*: Ka/Ks = 0.36441, JKa/JKs = 0.26757) (Figure 2; Table S4), indicating that they are the most conservative genes among 13 PCGs. The evolutionary rates of these genes in Ichneumonidae are similar to other lineages, such as Apiodea [15,16].

### 3.3. Gene Rearrangement

**Figure 3:**
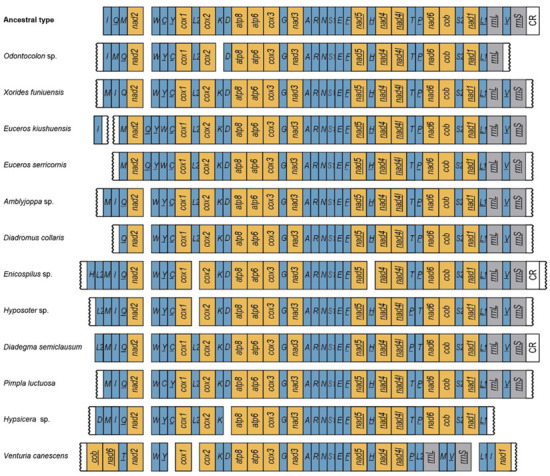
Mitochondrial genome rearrangement patterns of Ichneumonidae referenced with the ancestral insect mitochondrial genomes. PCGs, tRNA genes, rRNA genes and A+ T-rich region (CR region) are marked in orange, blue, grey and white, respectively. tRNA genes are denoted by a one-letter symbol according to the IPUC-IUB single-letter amino acid codes. Genes with underlines indicate that the gene is encoded on the minority strand. Genes with squiggle lines means the breakpoint caused by failure of sequencing. *cox1-cox3*: cytochrome oxidase subunits; *cob*: cytochrome b; *nad1-nad6*: NADH dehydrogenase com- ponents; *rrnL* and *rrnS*: ribosomal RNAs. Single letters identify the transfer RNA genes—refer to the IPUC-IUB (International Union of Pure and Applied Chemistry-International Union of Biochemistry) single-letter amino acid codes.

Compared with the putative ancestral mitochondrial genome of insects, no rearrangement events of PCGs appeared in the four newly sequenced mitochondrial genomes, which is consistent with what is observed from other species in Ichneumonidae, except for *Venturia canescens*. Both species of Eucerotinae and Xoridinae have different tRNA rearrangements. In Xoridinae, as most species in Ichneumonidae, the *trnW-trnC-trnY* cluster of *Odontocolon* sp. and *Xorides funiuensis* is shuffled as *trnW-trnY-trnC* (Figure 3). The *trnI-trnQ-trnM* cluster also has a significant shuffling, with *Odontocolon* sp. Shuffling as *trnI-trnM-trnQ*, while *Xorides funiuensis* is shuffled as *trnM-trnI-trnQ*, and *trnQ* is inversed (Figure 3). There is a remote translocation of *trnK* in cluster *trnK-trnD,* with *trnK* failed to be assembled in *Odontocolon* sp. (Figure 3). In Eucerotinae, both *Euceros* species share the same tRNA rearrangement events, except that *trnI* is not detected in *Euceros serricornis*. Gene *trnQ* transfers to the adjacent cluster *trnW-trnC-trnY* with the shuffling of *trnY*, resulting in a larger block *trnQ-trnY-trnW-trnC*. All the gene arrangement events of the four newly sequenced mitogenomes are unique and were not reported before in Ichneumonidae.

### 3.4. Phylogenetic Relationships within Ichneumonidae Based on Mitochondrial Genomes

The phylogenetic topologies were reconstructed using 2 phylogenetic Algorithms (Bayesian and maximum likelihood inference) based on 13 PCGs from 12 complete or partial mitochondrial genomes, representing 7 subfamilies of Ichneumonidae.

**Figure 4:**
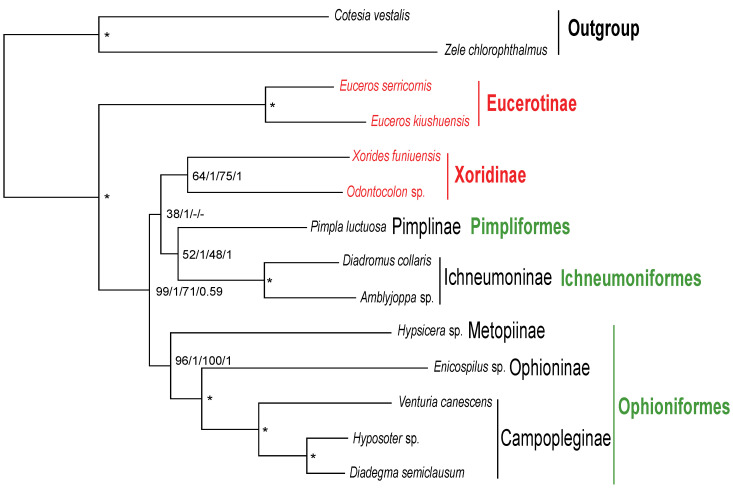
Phylogenetic relationships of family Ichneumonidae inferred from datasets nucleotides (NU) and amino acids (AA) sequences of 13 protein-coding genes in mitochondrial genomes using BI and ML methods. *Zele chlorophthalmus* and *Cotesia vestalis* from Braconidae were employed as outgroup. The numbers separated by “/” indicate the posterior probability (PP) and bootstrap values (BS) of the corresponding nodes from left to right: PP of NU, BS of NU, PP of AA, BS of AA. “*” indicates that the node was fully supported by all four inferences.

All the analyses recovered the three main high-level groupings, Pimpliformes, Ichneumoniformes and Ophioniformes and the topology of ((Pimpliformes + Ichneumoniformes) + Ophioniformes), which are uncontroversial with current opinions [6,7,8]. Eucerotinae was suggested as the basal lineage of Ichneumonidae with PP = 1 and BS = 100 in all analyses (Figure 4; Figures S1–S4), which represents a new basal ichneumonid lineage. Eucerotinae were clustered within Tryphoninae near to Ophioniformes [9] or closely related with Ichneumoformes [7,8,10,11] in previous studies. The position of Xoridinae within Ichneumonidae was still not determined in our study. Xoridinae is the sister group of Pimplinae and Ichneumoninae in the analysis based on an AA dataset of 13 protein-coding genes, as proposed by Bennett et al. [8] (Figure 4; Figures S1 and S2). Meanwhile the NU dataset indicates that it is the sister to eight other species (excluding Eucerotinae), which is supported by some previous studies [8,9,12] (Figures S3).

## 4. Conclusions

Using next-generation sequencing, four mitochondrial genomes of *Odontocolon* sp., *Xorides funiuensis*, *Euceros kiushuensis* and *Euceros serricornis* were presented. Their mitogenome organizations, gene rearrangement patterns and phylogenetic relationships with the other species from Ichneumonidae were analyzed. Limited gene arrangements appeared in all newly sequenced mitogenomes, however, all of them had novel rearrangement patterns of mitochondrial genomes in Ichneumonidae. In Xoridinae, *Odontocolon* sp. is shuffled as *trnI*-*trnM*-*trnQ* while *Xorides funiuensis* is shuffled as *trnM*-*trnI*-*trnQ,* and *trnQ* is inversed. There is a remote translocation of *trnK* in cluster *trnK*-*trnD* in *Odontocolon* sp. The two species of Eucerotinae shared a larger block *trnQ-trnY*-*trnW*-*trnC*. Both ML and BI analyses highly supported that Eucerotinae is the basal lineage for Ichneumonidae rather than Xoridinae. The relationship of Xoridinae with the other subfamilies was not determined. Given that the limited representatives of mitogenomes have been sequenced in each subfamily within Ichneumoninae, the results of the placements of Eucerotinae and Xoridinae would be altered if a denser taxon sampling was used, resulting in a more accurate and comprehensive tree that would better clarify the relationship within Ichneumonidae.

## Figures and Tables

**Table 1:** Taxonomic information, GenBank accession numbers and references of mitochondrial genomes used in the study.

Subfamily	Species	Accession Number	Reference
Ichneumoninae	*Amblyjoppa* sp.	MG923483	Tang et al. [15]
Ichneumoninae	*Diadromus collaris*	JX131613	Li et al. [35]
Ophioninae	*Enicospilus* sp.	FJ478177	Dowton et al. [19]
Campopleginae	*Venturia canescens*	FJ478176	Dowton et al. [19]
Campopleginae	*Hyposoter* sp.	MG923499	Tang et al. [15]
Campopleginae	*Diadegma semiclausum*	EU871947	Wei et al. [36]
Pimplinae	*Pimpla luctuosa*	MG923506	Tang et al. [15]
Metopiinae	*Hypsicera* sp.	MG923500	Tang et al. [15]
Xoridinae	*Odontocolon* sp.	MT252850	This study
Xoridinae	*Xorides funiuensis*	MT252851	This study
Eucerotinae	*Euceros kiushuensis*	MT252852	This study
Eucerotinae	*Euceros serricornis*	MT252853	This study
Euphorinae (outgroup)	*Zele chlorophthalmus*	MG822749	Direct Submission
Microgastrinae (outgroup)	*Cotesia vestalis*	FJ154897	Wei et al. [1]

**Table 2:** Base composition of the mitochondrial genomes in Ichneumonidae.

Species	Sequences Length (bp)	Protein-Coding Genes (PCGs)			
		(A + T) %	AT-Skew	GC-Skew	Length (bp)
*Amblyjoppa* sp.	17,110	80.07	−0.1113	−0.0687	11,097
*Diadromus collaris*	14,621	82.86	−0.1147	−0.0421	11,088
*Enicospilus* sp.	15,300	84.01	−0.1118	0.0330	11,175
*Venturia canescens*	13,635	84.01	−0.1277	−0.0050	10,008
*Hyposoter* sp.	18,893	83.45	−0.1200	−0.0227	11,202
*Diadegma semiclausum*	18,728	83.76	−0.1205	−0.0039	11,166
*Pimpla luctuosa*	16,926	81.11	−0.1210	−0.0258	11,088
*Hypsicera* sp.	17,017	78.26	−0.1431	−0.0412	11,175
*Odontocolon* sp.	13,321	78.60	−0.1009	−0.0521	11,199
*Xorides funiuensis*	14,939	82.35	−0.1180	−0.0214	11,130
*Euceros kiushuensis*	16,331	83.99	−0.1627	0.0782	11,097
*Euceros serricornis*	15,787	83.53	−0.1581	0.0537	11,088

## Data Availability

The new mitogenome assemblies and annotation data in this study have been submitted to the GenBank database under accession numbers: MT252850-MT252853.

## Supplementary Materials

The following are available online at https://www.mdpi.com/article/10.3390/genes13020218/s1, Figure S1: Phylogenetic relationships of Ichneumonidae inferred from nucleotides (NU) sequences of 13 protein-coding genes in mitochondrial genomes by BI methods; Figure S2: Phylogenetic relationships of Ichneumonidae inferred from nucleotides (NU) sequences of 13 protein-coding genes in mitochondrial genomes by ML methods; Figure S3: Phylogenetic relationships of Ichneumonidae inferred from amino acids (AA) sequences of 13 protein-coding genes in mitochondrial genomes by BI methods; Figure S4: Phylogenetic relationships of Ichneumonidae inferred from amino acids (AA) sequences of 13 protein-coding genes in mitochondrial genomes by ML methods. Table S1: Information for the samples sequenced in this study; Table S2: Best model list from PartitionFinder for seven subsets including 39 PCGs partitions (every PCG gene was part to tree partitions as p1, p2 and p3) and two subsets including 13 AA partitions for MrBayes; Table S3: Best model list from ModelFinder for 39 individual PCGs partitions (every PCG gene was part to tree partions as p1, p2 and p3) and 13 AA partitions for IQtree: Table S4: Synonymous and nonsynonymous substitutional analysis of 13 protein-coding genes.
