# Supplementary material for: Mitochondrial Genomes Yield Insights into the Basal Lineages of Ichneumonid Wasps (Hymenoptera: Ichneumonidae)
Source: Genes (Basel). 2022 Jan 25;13(2):218. doi: 10.3390/genes13020218 (PMC8872219; doi:10.3390/genes13020218)
Supplement: Supplementary file 1 [file genes-13-00218-s001.zip › genes-1512674-supplementary.pdf]

Supplementary tables

Table S1 Information for the samples sequenced in this study.

Voucher specimens	Subfamily	Species	Sex	Collection Place
ZJUHW_202000017	Xoridinae	*Odontocolon* sp.	Female	Liaoning, China
ZJUHW_202000018	Xoridinae	*Xorides funiuensis*	Female	Sichuan, China
ZJUHW_202000019	Eucerotinae	*Euceros kiushuensis*	Female	Tibet, China
ZJUHW_202000020	Eucerotinae	*Euceros serricornis*	Female	Sichuan, China

Table S2 Best model list from PartitionFinder for seven subsets including 39 PCGs partitions (every PCG gene was part to tree partitions as p1, p2 and p3) and two subsets including 13 AA partitions for MrBayes.

Subset	Best Model	Partition Names
1	GTR+I+G	c3p1, a6p1, n3p1, c1p1, c2p1, cbp1
2	F81+G	n2p1, a8p1, n6p1
3	HKY+G	n4lp3, n5p3, n1p3, n4p3
4	GTR+I+G	n5p2, n1p2, n4lp2, n4p2, n2p2, a6p2, n3p2, a8p2, n6p2
5	HKY+G	c3p3, c1p3, c2p3, a6p3, cbp3, n2p3, n6p3, n3p3, a8p3
6	GTR+I+G	n1p1, n4lp1, n5p1, n4p1
7	GTR+I+G	c3p2, c1p2, c2p2, cbp2
1	MTREV+I+G	c1, n4, n5, cb, c3, a6, a8, c2, n4l, n1, n3, n6
2	MTREV+I+G	n2

Table S3 Best model list from ModelFinder for 39 individual PCGs partitions (every PCG gene was part to tree partions as p1, p2 and p3) and 13 AA partitions for IQtree.

Best Model	Partition Names
HKY+F+R2	n5p3, cbp3, n2p3, n6p3
HKY+F+G4	c1p3, n4lp3, a8p3, n1p3
GTR+F+G4	n4p2, c1p1, cbp1, n1p1
K3Pu+F+I+G4	n4lp1
F81+F+G4	n3p2, a8p2, n6p2
TVM+F+G4	cbp2, n4lp2, c3p1, c2p1, c2p2
GTR+I+G	cbp2, c1p2, c2p2, cbp2
GTR+F+I+G4	n5p1, n4p1, n5p2
TN+F+R2	c2p3
TIM+F+G4	n2p1
TN+F+G4	a6p3, c3p3
K3Pu+F+G4	c3p2, n6p1, n1p2, a6p2
K3Pu+F+R2	n3p3, a6p1, c1p2
F81+F+R2	a8p1
TIM2+F+G4	n3p1
TPM2+F+G4	n4p3
mtInv+G4	n4l, c3
mtART+F+G4	n4, a6, n3, n1, cb
mtInv+F+G4	n2
mtART+G4	c1, a8
mtZOA+F+G4	n6
mtART+F+I+G4	n5, c2

Table S4 Synonymous and nonsynonymous substitutional analysis of 13 protein-coding genes

Genes	Ka	Ks	Ka/Ks	JKa	JKs	JKa/JKs
atp6	0.23645	0.38139	0.61997	0.28589	0.54747	0.52220
atp8	0.38877	0.33497	1.16061	0.55786	0.47281	1.17988
cox1	0.11008	0.42749	0.25750	0.11948	0.65027	0.18374
cox2	0.17502	0.40122	0.43622	0.20143	0.58626	0.34358
cox3	0.19583	0.42471	0.46109	0.22817	0.64933	0.35139
cob	0.15948	0.43764	0.36441	0.18006	0.67294	0.26757
nad1	0.22266	0.33955	0.65575	0.26608	0.46157	0.57647
nad2	0.36600	0.33715	1.08557	0.52444	0.46304	1.13260
nad3	0.24033	0.39791	0.60398	0.29175	0.61191	0.47679
nad4	0.26307	0.31060	0.84697	0.32633	0.40722	0.80136
nad4l	0.28697	0.29076	0.98697	0.36973	0.37384	0.98901
nad5	0.27437	0.30554	0.89798	0.34405	0.39751	0.86551
nad6	0.33660	0.33786	0.99627	0.45254	0.46958	0.96371

Abbreviations: Ka, synonymous substitutional rates; Ks, nonsynonymous substitutional rates; JKa, synonymous substitutional rates using the Jukes and Cantor (1969) correction; JKs: nonsynonymous substitutional rates using the Jukes and Cantor (1969) correction.

Supplementary Figures S1–S4

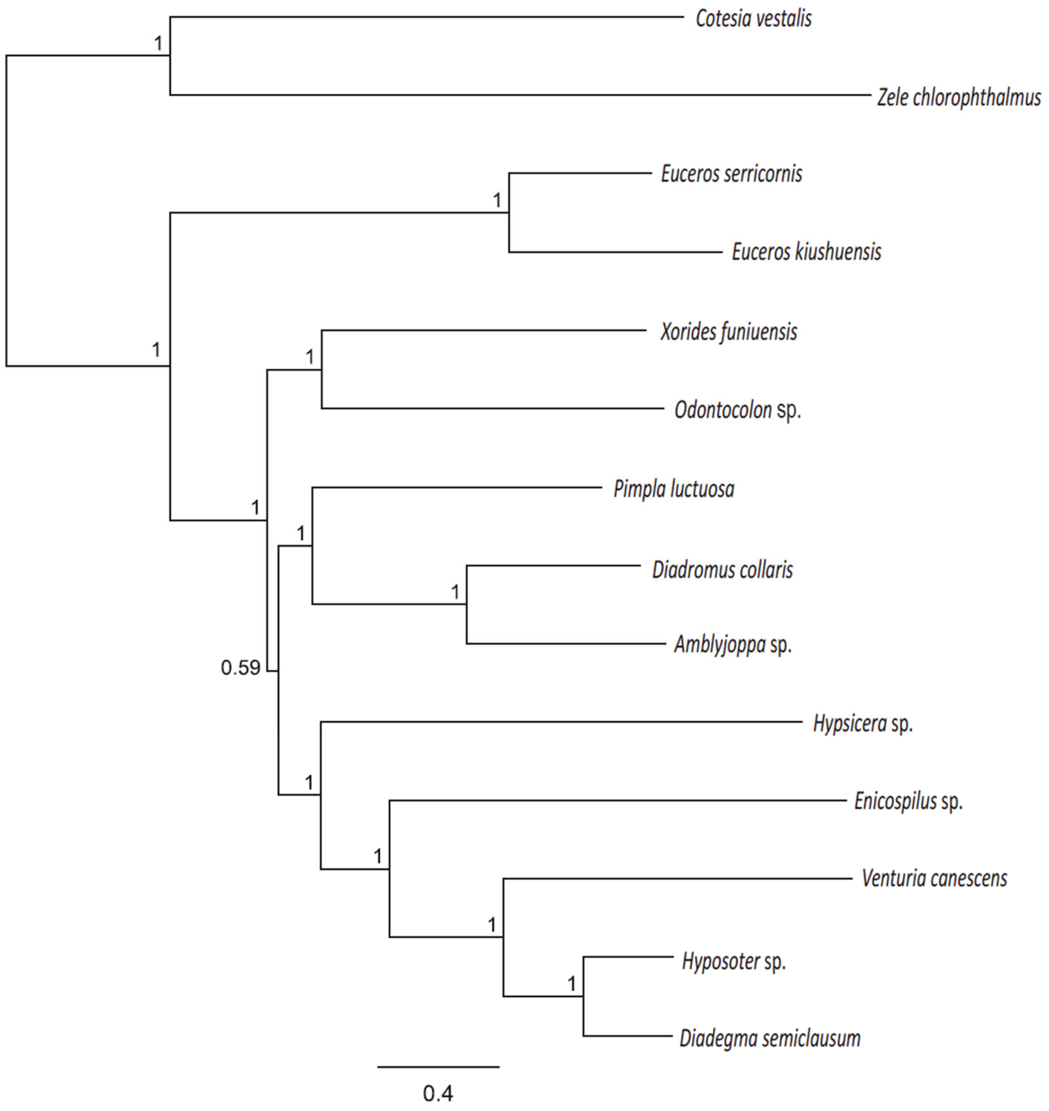

Figure S1 Phylogenetic relationships of Ichneumonidae inferred from nucleotides (NU) sequences of 13 protein-coding genes in mitochondrial genomes by BI methods. *Zele chlorophthalmus* and *Cotesia vestalis* from Braconidae were employed as outgroup. Numbers close to the branching points are Bayesian posterior probabilities (PP).

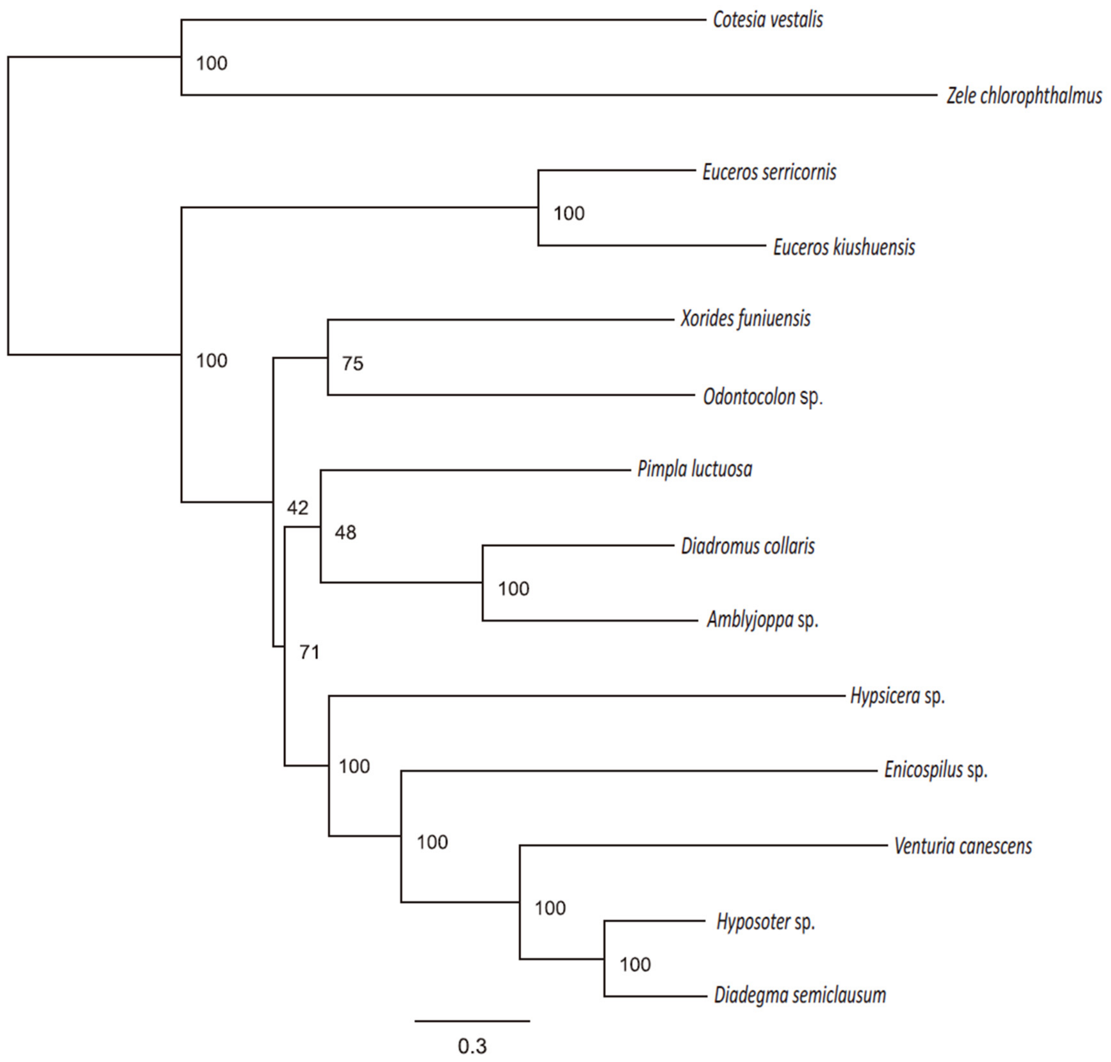

Figure S2 Phylogenetic relationships of Ichneumonidae inferred from nucleotides (NU) sequences of 13 protein-coding genes in mitochondrial genomes by ML methods. *Zele chlorophthalmus* and *Cotesia vestalis* from Braconidae were employed as outgroup. Numbers close to the branching points are bootstrap values (BS) calculated by maximum likelihood method.

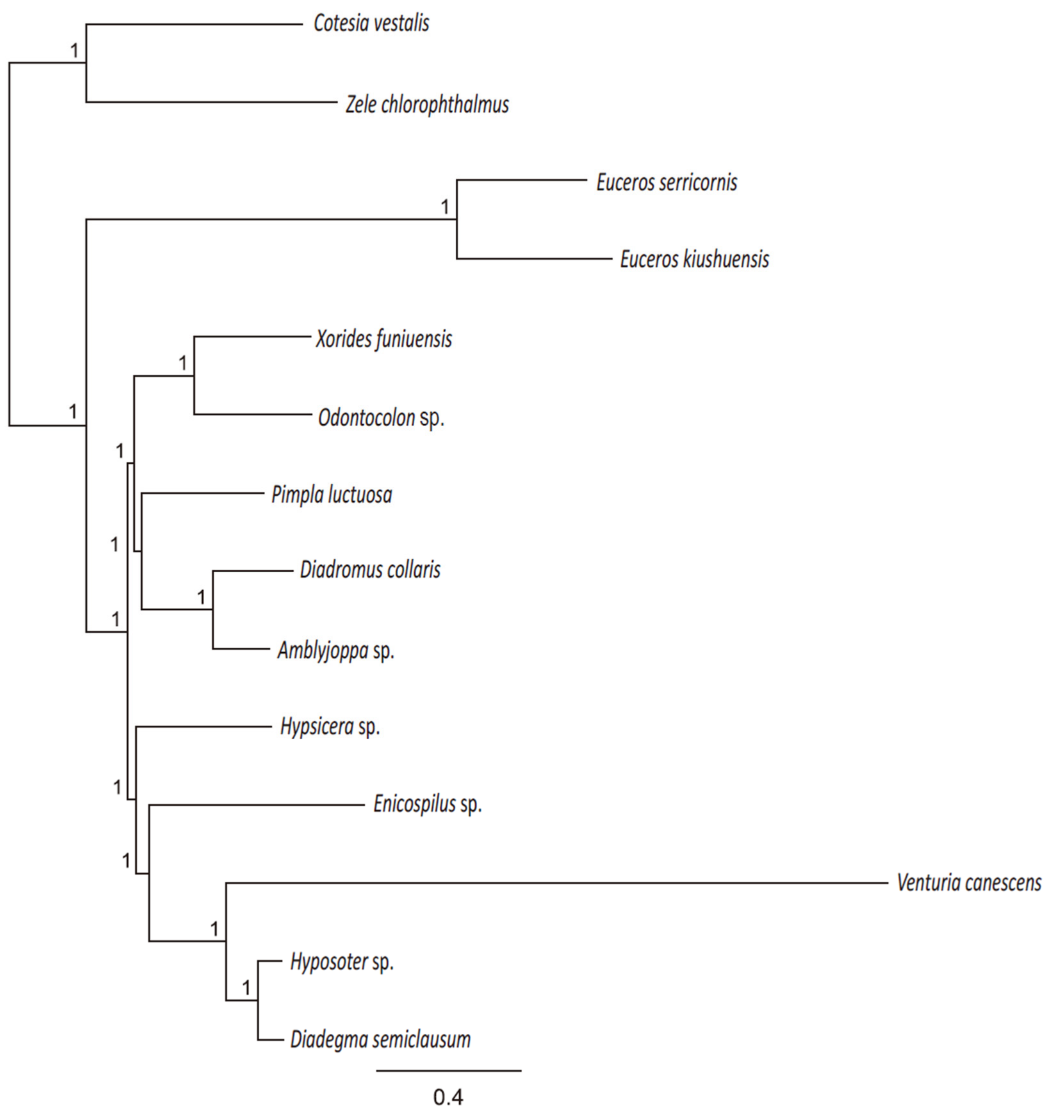

Figure S3 Phylogenetic relationships of Ichneumonidae inferred from amino acids (AA) sequences of 13 protein-coding genes in mitochondrial genomes by BI methods. *Zele chlorophthalmus* and *Cotesia vestalis* from Braconidae were employed as outgroup. Numbers close to the branching points are Bayesian posterior probabilities (PP).

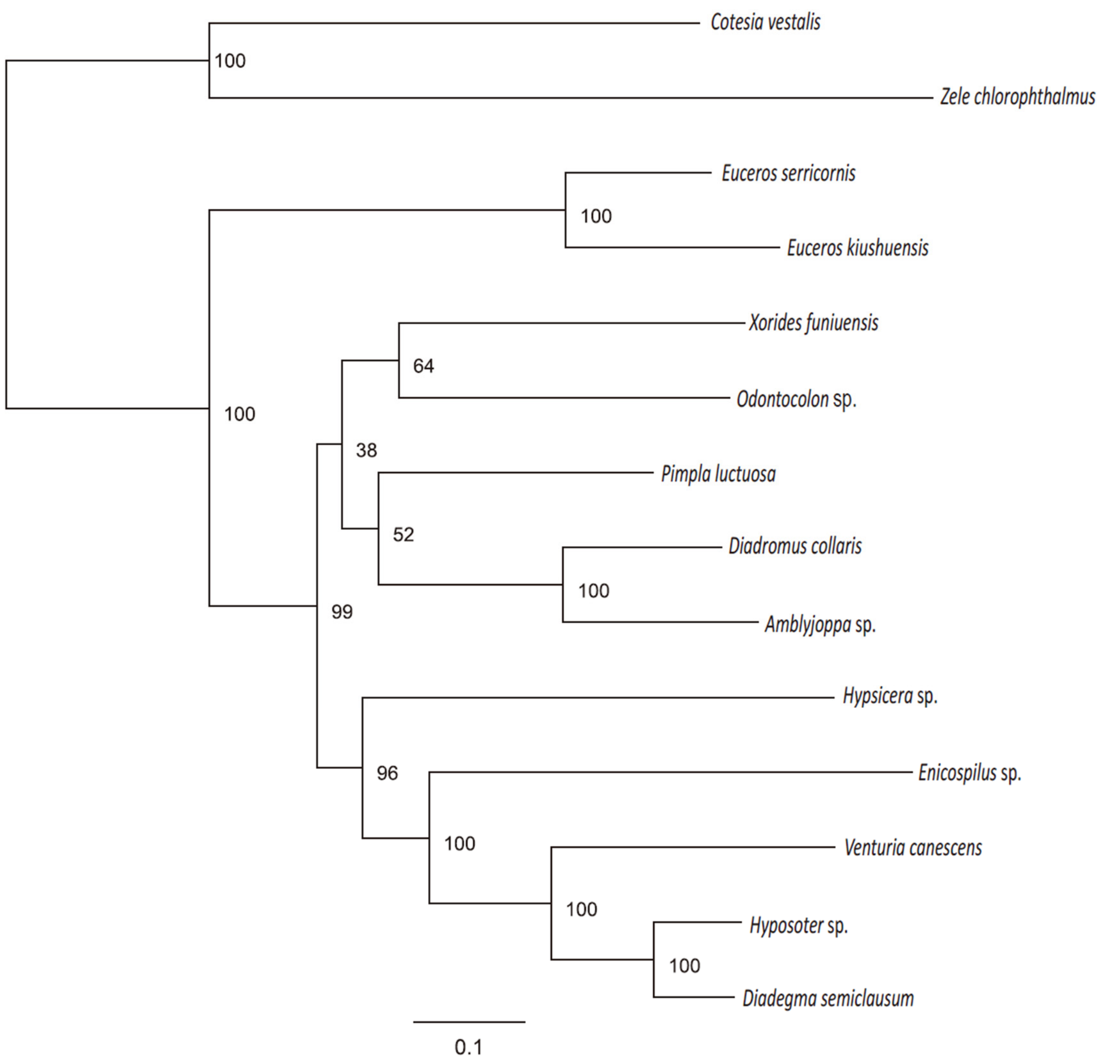

Figure S4 Phylogenetic relationships of Ichneumonidae inferred from amino acids (AA) sequences of 13 protein-coding genes in mitochondrial genomes by ML methods. *Zele chlorophthalmus* and *Cotesia vestalis* from Braconidae were employed as outgroup. Numbers close to the branching points are bootstrap values (BS) calculated by maximum likelihood method.
